# DNA methylation and copy number alterations in the progression of HPV‐associated high‐grade vulvar intraepithelial lesion

**DOI:** 10.1002/ijc.35366

**Published:** 2025-02-12

**Authors:** Flavia Runello, Aude Jary, Sylvia Duin, Yongsoo Kim, Kahren van Eer, Féline O. Voss, Nikki B. Thuijs, Maaike C. G. Bleeker, Renske D. M. Steenbergen

**Affiliations:** ^1^ Department of Pathology Amsterdam UMC Amsterdam The Netherlands; ^2^ Cancer Center Amsterdam Imaging and Biomarkers Amsterdam The Netherlands; ^3^ National Institute for Public Health and the Environment Centre for Infectious Disease Control Netherlands

**Keywords:** copy number alterations, DNA methylation, high‐grade vulvar intraepithelial lesion, mFAST‐SeqS

## Abstract

Human papillomavirus (HPV)‐associated high‐grade vulvar intraepithelial lesion (HSIL) is a precursor of vulvar squamous cell carcinoma (VSCC). Because of the 8% cancer risk, many vulvar HSIL patients undergo aggressive and mutilating treatments. Characterizing HSIL by their progression risk can help individualize treatment strategies. Accordingly, copy number alterations (CNAs) and DNA methylation have been identified as biomarkers for cancer risk stratification of HSIL. Here, we assessed their potential correlation, and relation to HPV16 (sub)lineages and progression to vulvar cancer. Eighty‐two vulvar formalin‐fixed paraffin‐embedded (FFPE) samples, including controls, HSIL, HSIL adjacent to VSCC and VSCC, with previously determined DNA methylation profiles, were analysed for CNAs using mFAST‐SeqS. Genome‐wide *z*‐scores were calculated to determine overall aneuploidy (aneuploidy scores), and compared to the methylation levels and status of marker panel *ZNF582/SST*/*miR124‐2*. For 52 HPV16‐positive cases, HPV (sub)lineages were determined by Sanger sequencing. HPV16 lineage A was predominant (86.4%), followed equally by lineages B, C, and D. Frequent chromosomal alterations included chr1pq, chr3q, chr9q gains, and chr2q, chr4q losses. Median aneuploidy scores increased across disease categories, from 0 in controls, to 3 in HSIL, 16 in HSIL adjacent to VSCC and 29 in VSCC. A positive relationship between aneuploidy scores and DNA methylation levels was found (*ρ* = 0.61, Spearman's rank correlation test). Aneuploidy scores were significantly higher in methylation‐positive samples (*p* < .001). In conclusion, we showed that DNA methylation and CNAs both rise with increasing severity of disease, indicating their prognostic value for cancer risk stratification of HSIL, while no relation to HPV16 (sub)lineages was found.

## Graphical abstract

What's new?

Patients with human papillomavirus (HPV)‐associated high‐grade vulvar intraepithelial lesion (HSIL) often undergo damaging treatments to prevent progression to cancer. Molecular characterization of HSIL to assess progression risk could reduce the need for aggressive interventions. Here, cancer risk stratification of HSIL was investigated by copy number alterations (CNAs) and DNA methylation analysis in vulvar specimens. Analyses reveal a positive relationship between CNAs and DNA methylation, wherein both increase with progression to vulvar cancer. HPV16 lineages, which influence viral oncogenicity, had no relationship with progression to cancer. The novel insights underscore the prognostic potential of CNAs and DNA methylation for cancer risk stratification in HSIL patients.

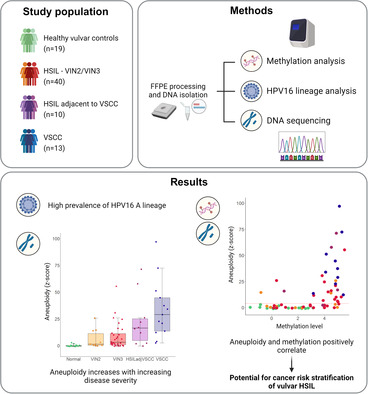

## 1 INTRODUCTION

Vulvar squamous cell carcinoma (VSCC) arises from precursor high‐grade vulvar intraepithelial neoplasia (VIN) in the vast majority of cases. VIN is currently categorized into human papillomavirus (HPV)‐associated high‐grade squamous intra‐epithelial lesion (HSIL), which includes vulvar intraepithelial neoplasia grade 2 and grade 3 (VIN2 and VIN3), and HPV‐independent VIN.^1^ The process of high‐risk human papillomavirus (hrHPV)‐mediated vulvar carcinogenesis represents a relevant biological and clinical subject, given the relatively low cancer risk of vulvar HSIL (8% in 10 years),^1^ the possibility for HSIL regression and the occurrence of multiple HPV‐related lesions within the anogenital tract, which has impact on patient treatment and morbidity.^2^

It is widely recognized that hrHPV drives carcinogenesis through the overexpression of viral early genes E6 and E7. In the context of cervical cancer, comprehensive studies have shown that different HPV16 lineages can influence virus oncogenicity, resulting in varying risks of disease and cancer progression: non‐European lineages B/C/D have been described as more pathogenic than the European lineage A.^3^, ^4^, ^5^, ^6^ The E6 and E7 oncoproteins disrupt many cellular processes, including the function of cell‐cycle and DNA damage regulators, through inactivation of host cell tumour suppressors p53 and retinoblastoma protein (pRb). Counteracting tumour suppressors during HPV‐induced carcinogenesis is not only achieved through direct binding at the protein level, but also at the DNA level, through epigenetic regulation.^7^, ^8^ In particular, DNA methylation of cellular genes is a signature of hrHPV‐mediated carcinogenesis, often inducing silencing of tumour suppressor genes. This process has been reported to occur already in precursor stages of disease. For this reason, DNA methylation is considered as an established biomarker for cancer risk stratification of precursor lesions in HPV‐related malignancies.^9^, ^10^, ^11^ Accordingly, a previous study has identified several genes whose methylation levels show an increase with increasing vulvar disease severity.^12^ DNA methylation was tested across different vulvar disease categories: healthy vulvar tissue, HSIL/VIN2 and HSIL/VIN3, HSIL adjacent to VSCC, recognized as a surrogate of the most advanced premalignant stage,^13^ and VSCC.^12^ The diagnostic performance of these promising methylation markers for VIN detection has been validated in a large series of both HSIL and HPV‐independent VIN lesions.^14^

Other than promoting cell proliferation through disruption of p53 and pRb activity, E6 and E7 expression also impacts cellular genome integrity, facilitating genomic instability.^15^ Genomic instability, an enabling hallmark of cancer,^16^ includes structural and numerical chromosomal alterations, resulting in gains and/or losses of specific chromosomal regions as well as entire chromosomal arms. These alterations, known as copy number alterations (CNAs), have been recorded in precursor lesions of HPV‐related cancers of the anogenital tract.^13^, ^15^, ^17^, ^18^, ^19^, ^20^ In HPV‐associated VSCC, frequently reported CNAs include gains of chromosome arms 1q, 3q, and 8q, and losses of chromosomes arms 3p, 8p, and 11q.^15^, ^21^, ^22^, ^23^ Research on CNAs in HSIL is limited.^13^, ^24^ Shallow sequencing analysis of a well‐characterized series of HSIL and VSCC tissues identified chromosome arms 1pq gain as a robust marker for cancer risk prediction, as well as a general higher number of CNAs in HSIL of patients that would later develop VSCC.^13^ However, no other studies have investigated and analysed a potential correlation between both genetic and epigenetic alterations in vulvar disease and VSCC. We have recently validated the modified fast aneuploidy screening test‐sequencing system (mFAST‐SeqS), based on the amplification of LINE‐1 sequences, as a reliable targeted sequencing method to identify CNAs in small formalin‐fixed paraffin‐embedded (FFPE) samples. In this study, mFAST‐SeqS analysis on anal and vulvar cancer precursor lesions has yielded results in line with conventional shallow‐sequencing analysis and previous findings.^24^

In order to compare the contribution of epigenetic events and genetic ones during vulvar carcinogenesis, the current study aimed at investigating HPV16 (sub)lineages and the copy number profiles of a series of 50 vulvar premalignant lesions and 13 VSCC using mFAST‐SeqS. DNA copy number profiles were next compared to previously determined methylation profiles to assess the potential correlation between both cancer‐driving molecular processes.

## 2 MATERIALS AND METHODS

### 2.1 Tissue samples

Copy‐number and methylation analyses were performed on 82 FFPE tissue samples of 63 HPV‐positive vulvar lesions, including 40 HSIL (10 VIN2, 30 VIN3), 10 HSIL adjacent to cancer (HSIL adjacent to VSCC), 13 vulvar squamous carcinomas (VSCC), and 19 HPV‐negative healthy vulvar tissue controls collected from patients undergoing aesthetic or reconstructive genital procedures. Six out of 40 HSIL (1 VIN2 and 5 VIN3) progressed to VSCC during follow‐up. We defined progression as a diagnosis of VSCC >3 months after HSIL, according to previous literature.^25^ The timespan between HSIL diagnosis and VSCC onset was 9 years for the patient with VIN2, and 4 months, 2, 9, 12, and 20 years for the VIN3 patients. FFPE samples were selected based on histology from prior studies, and collected as previously reported.^1^, ^13^ Two samples, one HSIL adjacent to VSCC and one VSCC, were collected from the same patient.

### 2.2 Tissue processing and molecular analysis

Retrieved FFPE tissue blocks were sectioned using the sandwich method: the first and last sections were stained with haematoxylin–eosin (H&E), while in‐between sections were cut for DNA isolation and subsequent molecular analysis.

Human papillomavirus (HPV) status and DNA methylation results were determined in our prior studies.^1^, ^13^ In short, immunohistochemical staining of p16^INK4a^ followed by hrHPV DNA‐testing were employed to determine the HPV status of each sample. Methylation analysis was performed by quantitative methylation‐specific polymerase chain reaction (qMSP), and results from the optimal methylation marker panel ZNF582, SST, and miR124‐2 (based on a former series of high‐grade VIN lesions (*n* = 580) and vulvar healthy controls (*n* = 113)^14^) were used in the present study.

### 2.3 HPV16 Sanger sequencing and lineage analysis

HPV16 variants were analyzed by amplification of the E6 and LCR regions followed by Sanger sequencing, as previously described.^26^ Briefly, four sets of outer primers were used to produce four PCR products of approximately 500 base‐pair (bp) length. Afterwards, nested PCR was performed using internal primer sets, leading to seven PCR products sizing from 211 to 350 bp. Primers sequences and detailed PCR protocols are reported in Tables S1 and S2. Sanger sequencing was performed by Macrogen company (https://dna.macrogen-europe.com).

The reconstruction of E6 and LCR sequences was conducted on the Geneious software. First, the seven fragments of each strain were mapped to the reference genome of HPV16 (NC_001526), to generate a consensus sequence. Then, multiple alignment of the new sequences was performed with Mafft and a set of 15 published sequences available on PaVE (https://pave.niaid.nih.gov/explore/variants/variant_genomes) and the GenBank database, including different (sub)lineages of HPV16. Finally, phylogenetic analysis was performed using PhyML, the maximum likelihood method (best model: GTR + G) and 1000 bootstraps resampling.

### 2.4 mFAST‐SeqS analysis

A detailed description of the mFAST‐SeqS protocol and sequencing results of part of the samples (*n* = 24) have already been published.^24^ Library preparation was based on a nested, LINE‐1 targeting PCR system, and carried out as previously reported.^24^ Shortly, a first PCR (LINE‐1 PCR) was carried out to amplify LINE‐1 sequences present in the starting DNA, followed by a second PCR (Index PCR) to add sample‐specific barcodes before normalization, pooling, and sequencing. Library DNA size and concentrations were visualized using the Agilent Bioanalyzer, 7500 DNA kit (Agilent, Santa Clara, USA). Sequencing reads were trimmed using Trimmomatic with the parameters ‘LEADING:3’, ‘TRAINING:3’, ‘SLIDINGWINDOW:4:15’, ‘MILEN:36’, ‘HEADCROP:16’ and mapped to the human genome hg19 using Burrow's Wheeler Alignmer (BWA aligner). Chromosome arm‐specific *z*‐scores and genome wide *z*‐scores were calculated as described by Belic et al.,^27^ in which genome‐wide *z*‐scores are based on the squared sum of all chromosome arm‐specific *z*‐scores. For normalization of read counts on total reads per sample, we used mFAST‐SeqS results of 19 healthy vulvar controls, as described previously.^24^ Male chromosome arms (Yp and Yq) were excluded, as well as 13p, 14p, 15p, 21p, and 22p which have insufficient LINE1 elements to yield reliable results. To determine the presence of gains or losses of chromosome arms in a sample, our previously determined threshold of four,^24^ which corresponds to a *p*‐value of 6 × 10e−4, was used for calling. To assess the extent of aneuploidy within each sample, genome‐wide *z*‐scores were calculated and referred to as “aneuploidy scores”, and aneuploidy was determined using our previously defined threshold of four.^24^

### 2.5 Statistical analyses

Boxplots with a standard notation (hinges correspond to the 1st and 3rd quantiles, whiskers represent maximum and minimum values) were computed to display aneuploidy scores (i) across disease categories and (ii) between methylation‐positive and ‐negative samples for marker panel *ZNF582/SST/miR124‐2*. Differences between disease categories were assessed using the Kruskal–Wallis test, followed by pairwise post hoc Mann–Whitney *U* testing with Bonferroni correction for multiple comparisons to assess the statistical significance. Pairwise comparisons were done using the Wilcoxon rank‐sum test.

Methylation levels across different categories were visualized by determining boxplots of the log2‐transformed ΔΔCt ratios of each individual marker. Predicted probabilities of the combined marker panel *ZNF582/SST/miR124‐2* were calculated for each sample, using a previously developed logistic regression model. For this model a cohort of healthy vulvar controls (*n* = 113) was compared to high‐grade VIN cases (HSIL and HPV‐independent VIN) (*n* = 580), and a threshold to categorise samples into methylation‐positive or methylation‐negative was based on Youden's Index.^14^

Scatter plots were generated to visualize the relationship between each sample's overall degree of aneuploidy, defined as aneuploidy score and (i) *ZNF582/SST/miR124‐2* log odds (a continuous measure of the 3‐marker panel methylation level), and (ii) each individual marker's methylation level. The relationship was quantified by Spearman's coefficient (*ρ*) (Spearman's rank correlation test) and classified as moderate to strongly positive if *ρ* > 0.6, fairly positive if 0.6 < *ρ* < 0.3, and poorly positive to none if 0.2 < *ρ* < 0.^28^

Multiple linear regression and logistic regression analyses were performed to estimate whether the relationship between aneuploidy scores (continuous) and DNA methylation levels (continuous) or DNA methylation status (i.e., positive, negative) was statistically significant when adjusting for disease severity (i.e., controls, HSIL/VIN2, HSIL/VIN3, HSIL adjacent to VSCC, VSCC; and controls, HSIL [VIN2, VIN3, HSIL adjacent to VSCC combined], VSCC). Statistical analyses and data visualization were performed in R (v4.3.2), using packages stats (v4.3.2), ggpubr (v0.6.0), and ggplot2 (v3.5.0). *p* < .05 was considered statistically significant and then categorized as marginal evidence (.01 < *p* < .05), moderate evidence (.001 < *p* < .01) and strong evidence (*p* < .001).

## 3 RESULTS

**FIGURE 1:**
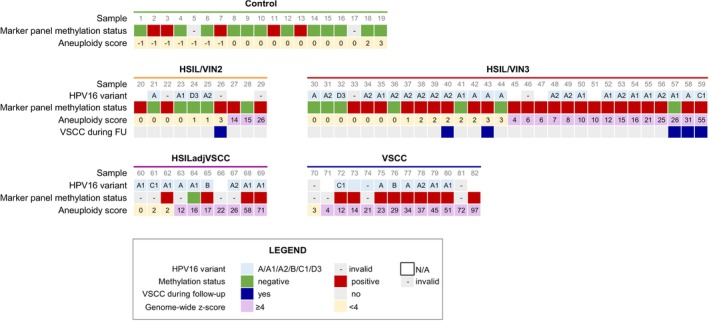
Overview of individual HPV16 variant, methylation status, aneuploidy scores and progression to VSCC per disease category. In each subgroup, samples are consecutively ordered low‐high based on their aneuploidy score. A, not assignable to any specific sublineage because of incomplete and/or absent LCR sequencing.

Through mFAST‐SeqS we obtained the copy number profiles of all 82 FFPE samples included in the analysis (number of sequenced reads is reported in Table S3). The samples comprised healthy vulvar tissues, HSIL (VIN2 and VIN3), six of which progressed to cancer during follow‐up, HSIL adjacent to VSCC and VSCC. Previously obtained results of hrHPV DNA‐testing and the DNA methylation marker panel *ZNF582, SST*, *miR124‐2* ^1^, ^13^ are summarized in Figure 1. Out of six HSIL that progressed to VSCC during follow‐up, five tested methylation‐positive and one (sample #57) tested methylation‐negative.

### 3.1 Distribution of HPV16 (sub)lineages across disease categories

A subset of 52 HSIL, HSIL adjacent to VSCC and VSCC samples positive for HPV16 were subjected to E6 and LCR Sanger sequencing (Table S4) to determine their lineages and sublineages. Sequencing was unsuccessful in eight samples, either because residual DNA was not available after molecular testing (*n* = 2) or because E6 and LCR amplification failed (*n* = 6). In the remaining 44 samples, the A lineage was represented the most (86.4%, 38/44), with 34.1% (15/44) belonging to sublineage A1, and 31.8% (14/44) to sublineage A2. The remaining 20.5% (9/44) was not assignable to any specific A sublineage, because of incomplete and/or absent LCR sequencing. Six samples (13.6% 6/44) belonged to either the B lineage (4.5%, 2/44), or sublineages C1 (4.5%, 2/44) or D3 (4.5%, 2/44). Phylogenetic trees are shown in Figure S3.

The distribution of HPV16 variants across the different HSIL lesions and VSCC is shown in Figure 1. The A lineage, specifically A1 and A2 sublineages, was present through all lesion types. Sublineage D3 was only present in HSIL (VIN2 and VIN3), sublineage C1 in HSIL/VIN3 and HSIL adjacent to VSCC, and the B lineage in HSIL adjacent to VSCC and cancers. Out of the six HSIL lesions that progressed to cancer during follow‐up, five had a valid sequencing result: one belonged to the C1 sublineage, while the other four belonged to the A lineage.

### 3.2 Copy number profiling shows a rise of genomic instability across disease categories

Copy number analysis by mFAST‐SeqS showed no chromosomal alterations in healthy vulvar controls, except for a gain on chromosome arm 1p in a single sample. Frequent alterations in other disease categories (>20% of samples) included gains on chromosome arms 1p, 1q, 3q, and 9q, and losses on chromosome arms 2q and 4q (Table 1). The median [IQR] number of gains detected with mFAST‐SeqS was 0.5 [0–2] in HSIL/VIN2, 1.5 [0–5] in HSIL/VIN3, 4 [1–5] in HSIL adjacent to VSCC and 6 [4–7] in VSCC, while the median [IQR] number of losses was respectively 0 [0–1.5], 0 [0–2], 2 [0–4], and 5 [3–8].

**TABLE 1:** Marker panel methylation status, most frequent copy number alterations (CNAs) and median aneuploidy score in different vulvar disease categories and VSCC.

	Methylation status (*n*)			Gains (*n*)				Losses (*n*)		Median aneuploidy score
	MM+	MM−	Invalid	1p	1q	3q	9q	2q	4q	
Control (*n* = 19)	5	12	2	1	0	0	0	0	0	0
HSIL/VIN2 (*n* = 10)	5	5	0	1	2	2	3	1	3	1
HSIL/VIN3 (*n* = 30)	23	7	0	13	11	7	9	4	7	3
HSILadjVSCC (*n* = 10)	4	1	5	4	7	7	1	4	3	16
VSCC (*n* = 13)	9	0	4	3	2	5	4	8	6	29
% Total	56.1	30.5	13.4	26.8	34.1	25.6	20.7	20.7	23.2	

Abbreviations: HSIL, high‐grade vulvar intraepithelial lesion; HSILadjVSCC, HSIL adjacent to VSCC; MM, methylation marker panel, VIN2, vulvar intraepithelial neoplasia grade 2; VIN3, vulvar intraepithelial neoplasia grade 3; VSCC, vulvar squamous cell carcinoma.

**FIGURE 2:**
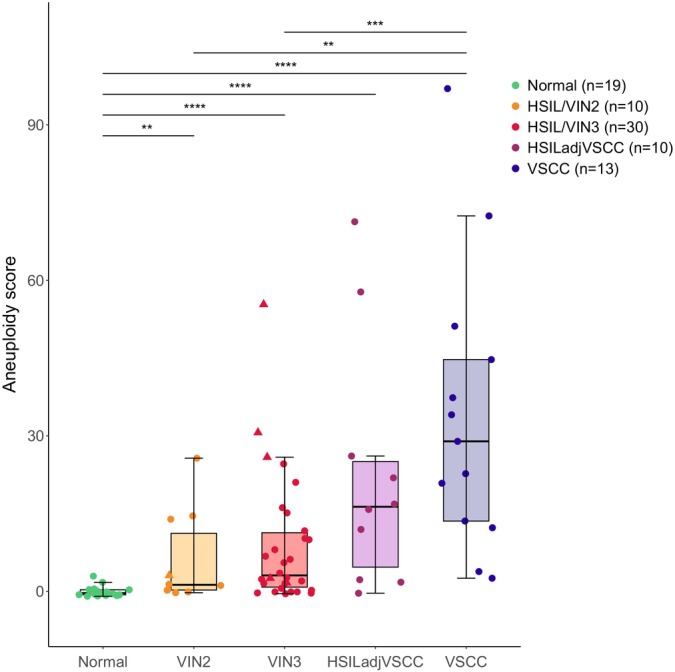
Aneuploidy scores levels across vulvar disease categories. Number of sequenced samples is reported in the legend. In the boxplots, hinges correspond to the 1st and 3rd quantiles, whiskers “min” and “max” correspond to 1.5*IQR, horizontal lines indicate the median. Triangles represent HSIL patients who developed VSCC during follow‐up. ns, not significant; *p < .05; **p < .01; ***p < .001; ****p < .0001.

As a general trend, aneuploidy scores (calculated as genome‐wide *z*‐scores) increased with the severity of the disease (Figure 2). Healthy controls showed a median aneuploidy score [IQR] of 0 [0–0], indicative of a minimal if not absent number of CNAs detectable with mFAST‐SeqS. The median aneuploidy score [IQR] rose to 1 [0–11] in HSIL/VIN2 and 3 [1–11] in HSIL/VIN3. A notable increase was detected in HSIL adjacent to VSCC with a median aneuploidy score [IQR] of 16 [5–25], followed by the highest values in cancers with a median of 29 [14–45]. Median aneuploidy scores are shown in Table 1. All disease categories had significantly higher aneuploidy scores when compared to the control group (Figure 2). Significant differences were also found between HSIL (VIN2 and VIN3) and cancers. Among the six samples collected from patients that developed VSCC during follow‐up, indicated by a triangle in Figure 2, three HSIL/VIN3 displayed aneuploidy scores comparable to some of the highest ones in HSIL adjacent to VSCC and VSCC categories (aneuploidy scores 26, 31, 55; samples #57, #58, #59, Figure 1). The remaining one HSIL/VIN2 and two HSIL/VIN3 showed lower aneuploidy scores of, respectively, 3, 3, and 2.

Calculation of the sum of CNAs per chromosome arm showed a very similar trend across disease categories as the aneuploidy scores (Figure S1).

### 3.3 Correlation between aneuploidy scores and DNA methylation levels

**FIGURE 3:**
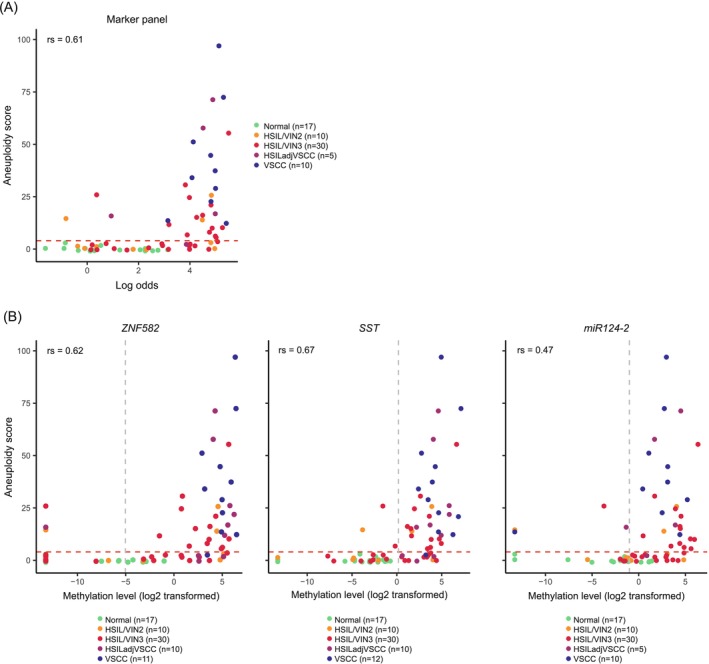
Scatter plots representing the relationship between DNA methylation and aneuploidy scores in different vulvar disease categories. (A) Comparison between methylation levels of combined marker panel *ZNF582/SST/miR124‐2* (calculated as log odds, see reference^14^) and aneuploidy scores of different vulvar disease categories. Number of methylation‐valid cases is reported in the legend. (B) Comparison between methylation levels of individual markers *ZNF582, SST, miR124‐2*, and aneuploidy scores of different vulvar disease categories. Number of methylation‐valid cases is reported in the legend. Samples reaching the maximum number of threshold cycles (ct) during qMSP resulted in a log2‐transformed ΔΔCt ratio of −13. Thresholds to categorize samples into methylation‐negative or methylation‐positive are represented by the grey dotted lines: 0.89 for marker panel *ZNF582/SST/miR124‐2*, −5.04 for *ZNF582*, 0.17 for *SS*T, and −1.00 for *mir124‐2*. Red dotted lines represent the threshold of four used as a measure of aneuploidy.

**FIGURE 4:**
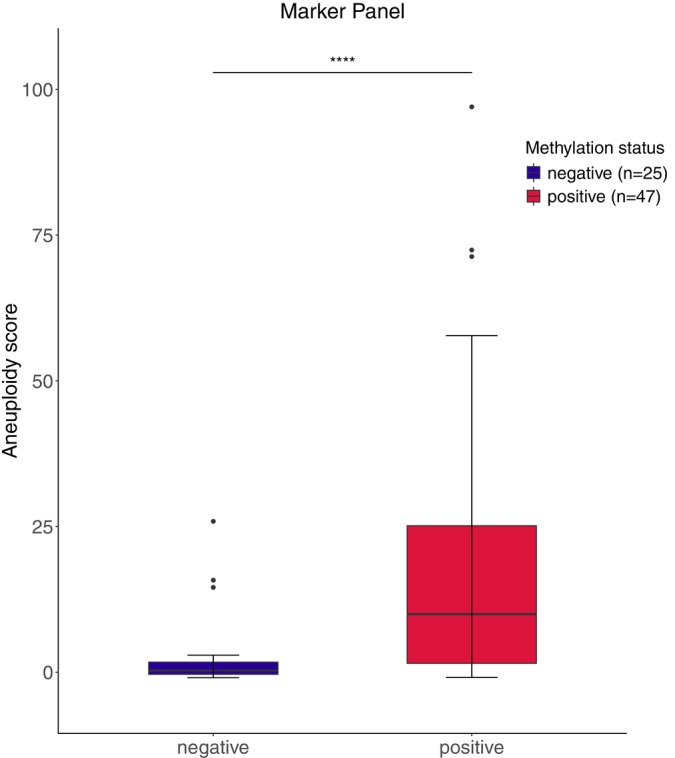
Aneuploidy scores in *ZNF582/SST/mir124‐2* methylation‐positive versus methylation‐negative patients. In the boxplots, hinges correspond to the 1st and 3rd quantiles, whiskers “min” and “max” correspond to 1.5*IQR, horizontal lines indicate the median. Outliers are represented by a black circle. ns, not significant; *p < .05; **p < .01; ***p < .001; ****p < .0001.

Next, we investigated the correlation between aneuploidy scores and previously obtained DNA methylation profiles, across the different disease categories. The methylation levels of each marker are shown in Figure S2. The methylation levels of all three markers were available for 72 samples and, combined into marker panel *ZNF582/SST/miR124‐2*, methylation levels increased with increasing aneuploidy scores across disease categories (Figure 3A), with a moderate to strong positive correlation (*ρ* = 0.61, Spearman's rank correlation test). Depending on the availability of DNA methylation data, the methylation levels of single markers *ZNF582*, *SST*, and *miR124‐2* were compared to the aneuploidy scores of respectively 79, 78, and 72 samples (Figure 3B). Overall, DNA methylation levels of all three markers increased with increasing aneuploidy scores across disease categories. Focusing on each marker, we observed that *ZNF582* and *SST* both showed a moderate to strong positive correlation to aneuploidy scores (respectively, *ρ* = 0.62 and *ρ* = 0.68, Spearman's rank correlation test). *Mir124‐2* followed the same trend, albeit to a lesser extent in comparison to the other markers (*ρ* = 0.47). We then categorized samples into methylation‐positive or ‐negative using our previously validated threshold of marker panel *ZNF582/SST/miR124‐2*.^14^ We observed that 29.4% (5/17) healthy controls, 50% (5/10) HSIL/VIN2, 76.7% (23/30) HSIL/VIN3, 80% (4/5) HSIL adjacent to VSCC, and 100% (10/10) VSCC samples tested methylation‐positive (Table 1). Comparing these results to those obtained from mFAST‐SeqS analysis, significantly higher aneuploidy scores were found in methylation‐positive samples as opposed to methylation‐negative ones (Figure 4). Median values [IQR] reached 10 [2–25] in methylation‐positive samples, in contrast to 0 [0–2] in methylation‐negative ones.

Additional multiple linear regression and logistic regression analyses did not show a statistically significant impact of methylation levels or methylation status on aneuploidy scores, nor of aneuploidy scores on methylation levels or methylation status, when accounting for the contribution of increasing disease severity (*p* > .05).

## 4 DISCUSSION

In this study, we show a positive relationship between increasing DNA methylation and accumulation of chromosomal alterations across all vulvar samples. Most HSIL and VSCC tested positive for HPV16, and through Sanger sequencing we found a predominance of the HPV16 A lineage (including sublineages A1 and A2), which is mainly reported in European countries.^6^ Through mFAST‐SeqS, we identified common cancer driving alterations such as chromosome arms 1pq and 3q gain, in line with published literature.^15^, ^21^, ^22^, ^23^ Most notably, our results indicate that patients with a positive methylation test of marker panel *ZNF582*, *SST* and *miR124‐2* display a higher overall degree of aneuploidy when compared to methylation‐negative patients. The association between methylation and aneuploidy was not statistically significant when accounting for disease severity as a covariate. Nevertheless, the notion that an increased number of CNAs and elevated methylation levels are both associated to an escalating severity of disease, implies that these genetic and epigenetic events play an important promoting role in vulvar carcinogenesis. Additionally, as DNA methylation positivity was more frequent than CNAs abundance in HSIL, we hypothesize that methylation might occur as an earlier event in disease progression.

It is well known that cancer genetics and epigenetics influence each other during the process of carcinogenesis.^29^ The integration of genetic and epigenetic features, together with other omics, has allowed for better cancer characterization, paving the way to precision oncology.^30^ For breast cancer, it has been suggested that DNA methylation might be a better cancer risk biomarker than copy number variations (CNVs), based on its ability to significantly discriminate cancer‐adjacent normal and healthy normal tissues.^31^ A similar conclusion has been drawn in the context of ovarian cancer, where DNA methylation has been found to better classify early‐stage ovarian carcinoma histotypes than CNAs.^32^ Nevertheless, the importance of combining multimodal data for a more comprehensive understanding of the biological and molecular processes driving carcinogenesis has been reiterated.^33^, ^34^ To our knowledge, this is the first study comparing DNA methylation patterns and genome‐wide copy‐number alterations in more than 80 vulvar samples with a thorough HPV genetic characterization, including a unique series of premalignant lesions that have progressed to cancer over the years.

Literature investigating HPV intra‐type genetic variants in HPV‐positive vulvar cancer and HSIL is, to the best of our knowledge, scarce. Similarities in HPV16 (sub)lineages distribution have been reported between vulvar and cervical cancers.^35^ Our results showed a predominance of HPV16 A (sub)lineages (A, A1, and A2) across different types of HSIL lesions and VSCC. This was not unexpected considering their reported global ubiquity, with lineage A being most common in Europe.^6^ Nevertheless, we also found a minority of samples harboring B, C1, and D3 (sub)lineages, which based on previous research on cervical cancer may be more pathogenic.^36^ Notably, (sub)lineages B and C1 were found in lesions testing almost completely methylation‐positive and with high aneuploidy scores. A large‐scale study on 3200 HPV16‐positive cervical samples found sublineage D3 to have a substantially higher risk for advanced CIN (CIN3) and cervical cancer.^36^ In our study, both lesions harboring sublineage D3 (one HSIL/VIN2 and one HSIL/VIN3) were methylation‐ and aneuploidy‐negative. indicating this sublineage might not be as pathogenic in vulvar carcinogenesis. However, in this context, our sample size only allows for speculation and this hypothesis would benefit from more extensive research.

DNA methylation has been reported as an early event in anogenital carcinogenesis.^37^ Contrarily, it has been harder to pinpoint at which stage chromosomal alterations occur.^15^ In their review,^38^ Trietsch et al. have reported from different studies a higher frequency of allelic imbalances in invasive vulvar carcinomas and advanced precursor lesions.^39^, ^40^ In 2016, Swarts et al. presented the same conclusion.^13^ Similarly, our results show a drastic increase of aneuploidy scores in HSIL adjacent to VSCC and VSCC samples. Moreover, a significant difference was also observed in HSIL when compared to healthy controls. While these findings clearly suggest that in vulvar carcinogenesis, the contribution of large‐scale genetic alterations begins in high‐grade premalignant stages of disease, it is worth pointing out the heterogeneity among samples in these disease categories. Looking at aneuploidy scores in HSIL, we can distinguish two groups: one with values as low as those of healthy controls, and one with values that are not only increased, but even comparable to those found in cancers. This heterogeneity is further proof of the challenge that is assigning an order to CNAs occurrence during carcinogenesis. Nevertheless, the percentage of cases belonging to the high aneuploidy scores group increased from HSIL/VIN2 to HSIL/VIN3, and only a minority of HSIL adjacent to VSCC and VSCC samples showed low aneuploidy scores. On the other hand, DNA methylation was more frequent even among those HSIL cases, especially VIN3, with lower aneuploidy‐scores; thus suggesting this epigenetic event may be among the first to characterize HSIL and its progression to VSCC. Accordingly, prior research on cervical cancer and CIN proposed that epigenetic events such as DNA methylation, and genetic ones such as chromosomal loss, contribute to carcinogenesis by cooperating in silencing specific genes; and methylation has been reported to occur before chromosomal loss.^41^

Previous studies have reported a remarkable diversity of CNAs in HSIL. Among the most commonly observed alterations, we find amplifications and deletions at chromosome arms 1pq, 3pq, 8q, and 11q, some of which have been associated to a higher risk of VSCC development.^15^, ^21^, ^22^, ^23^ Given this varied landscape of chromosomal alterations, an overall measure of aneuploidy with the same predictive value proves helpful. Accordingly, our results show that, in general, a higher burden of CNAs is related to a higher severity of disease. By using aneuploidy scores to quantify this rising aneuploidy, it is possible to assess the risk of disease progression through a single parameter. Moreover, obtaining this information using mFAST‐SeqS only requires a limited amount of DNA, which is ideal when FFPE material is scarce, and is economically convenient when compared to other methods such as Shallow sequencing.^24^ The mFAST‐SeqS technique derives from the original FAST‐SeqS, which was developed for non‐invasive fetal aneuploidy screening through maternal blood analysis.^42^ FAST‐SeqS was modified into mFAST‐SeqS as a pre‐screening tool to estimate cell‐free tumor DNA (ctDNA) percentage in plasma samples, thereby improving the accuracy of detecting of cancer‐related mutations in liquid biopsies.^43^ Although this adaptation of mFAST‐SeqS has shown great potential for clinical application,^44^, ^45^ its use for the analysis of FFPE material in routine diagnostics requires further standardization,^24^ limiting the swift translation of our findings to a clinical context. Another limitation concerns the nature of the sequencing method itself; by relying on the amplification of LINE‐1 sequences, mFAST‐SeqS is solely able to detect large‐scale chromosomal gains or losses. As a consequence, focal CNAs are not detected. However, the most relevant cancer‐driving alterations, that is, 3q and 1pq gain, were identified in, respectively, 30% (21/69), 30% (21/69), and 33% (23/69) of HSIL and VSCC samples. Additionally, in a previous study where mFAST‐SeqS failed to detect the occurrence of a 3q gain (confirmed by Shallow sequencing) in few HSIL samples, the same samples showed instead distinctively high aneuploidy scores.^24^ This suggests that, based on this value, mFAST‐SeqS is still able to provide reliable insight in the severity of the disease. Suitably, when considering those HSIL samples in our study in which alterations of chromosomes 3q and 1pq were not detected, two (one VIN2 and one VIN3) showed remarkable aneuploidy scores (14 and 26, respectively). Of note, the HSIL/VIN3 sample with an aneuploidy score of 26 was collected from a patient who developed VSCC during follow‐up. We hypothesize that the HSIL/VIN2 lesion with a high aneuploidy score might also be more at risk of progressing to vulvar cancer than others.

Six HSIL patients (one VIN2 and five VIN3) developed VSCC during follow‐up. The timespan between HSIL diagnosis and VSCC onset ranged from 4 months to 20 years, and the aneuploidy scores of these patients were either as high as those found in cancers (26, 31, and 55), or comparable to those found in some healthy controls (2, 3, and 3). While aneuploidy scores were inconsistent among these cases, methylation status was not: five lesions tested methylation positive. Notably, the one remaining methylation‐negative case had an aneuploidy score of 26. Based on our results indicating a positive relationship between CNAs and methylation, it would have been informative to investigate the methylation status of this patient at a time proximal to VSCC development, to determine whether methylation could have still played a decisive role in cancer progression, albeit later. Nevertheless, when looking at methylation in the other progressive cases, it was the patient with the highest *z*‐score who also displayed considerably higher methylation levels for all markers. Interestingly, we also identified one methylation‐positive VSCC case with an aneuploidy score of only 3, characterized by a single loss of chromosome arm 3p. Recent research on head and neck squamous cell carcinomas (HNSCC), also classified as HPV‐associated or ‐independent, has confirmed the existence of a subgroup of HNSCC that are CNA‐silent.^46^ Despite this subgroup being HPV‐independent, its genetic profile could provide an explanation for the exceptional case in our VSCC cohort.

In conclusion, we demonstrate that DNA methylation and CNAs correlate to disease severity in the process of HPV‐induced vulvar carcinogenesis. These molecular events, represented by DNA methylation of relevant tumor suppressor genes and an increased general level of cellular aneuploidy, are already detectable in high‐grade precursor lesions, indicating their prognostic value for cancer risk stratification of HSIL. No evidence was found for a potential role of HPV16 (sub)lineages. Based on our results, we hypothesize that DNA methylation might occur as an earlier event during premalignant stages of disease. While these notions implicate a form of complementarity between methylation and CNAs, additional prospective studies are required to explore this hypothesis and its clinical potential.

## AUTHOR CONTRIBUTIONS

**Flavia Runello:** Data curation; formal analysis; investigation; visualization; writing – original draft. **Aude Jary:** Data curation; formal analysis; investigation; writing – review and editing. **Sylvia Duin:** Data curation; investigation; writing – review and editing. **Yongsoo Kim:** Formal analysis; writing – review and editing. **Kahren van Eer:** Investigation; writing – review and editing. **Féline O. Voss:** Data curation; writing – review and editing. **Nikki B. Thuijs:** Investigation; writing – review and editing. **Maaike C. G. Bleeker:** Conceptualization; funding acquisition; writing – review and editing; supervision. **Renske D. M. Steenbergen:** Conceptualization; funding acquisition; methodology; writing – review and editing; supervision.

## FUNDING INFORMATION

This work was supported by the Dutch Cancer Society (KWF Kankerbestrijding; grant number 2016‐10382) and AJ was supported by the Fondation ARC pour la recherche sur le cancer.

## CONFLICT OF INTEREST STATEMENT

Renske D.M. Steenbergen is a minority shareholder of Self‐screen B.V., a spin‐off company of Amsterdam UMC, location VUmc. Self‐screen B.V. develops, manufactures and licenses high‐risk HPV and methylation marker assays and holds patents on these tests. Renske D.M. Steenbergen also declares consultancy fees from AstraZeneca. All the other authors declare that they have no competing interests.

## ETHICS STATEMENT

For the use of archived material we adhered to the Code of Conduct for Responsible Use of Left‐over Material of the Dutch Federation of Biomedical Scientific Societies and ethical approval was waived by the local Medical Ethics Committee under reference number 2017.561.

## Supporting information


**TABLE S1:** Primers sequences for the amplification of HPV16 E6 and LCR viral genome regions.
**TABLE S2:** PCR protocols for the amplification of HPV16 E6 and LCR viral genome regions.
**TABLE S3:** Number of total reads, total mapped reads, and median number of mapped reads per chromosomal arm*, per sample obtained after mFAST‐SeqS. Reads were mapped to the human genome hg19. *mFAST‐SeqS is a low‐resolution technique which performs analysis at chromosomal arm level.
**TABLE S4:** HPV16 variants in patients diagnosed with HSIL and VSCC lesions.
**FIGURE S1:** Total number of CNAs per sample across vulvar disease categories. Number of sequenced samples is reported in the legend. In the boxplots, hinges correspond to the 1st and 3rd quantiles, whiskers “min” and “max” correspond to 1.5*IQR, horizontal lines indicate the median. Triangles represent HSIL patients who developed VSCC during follow‐up. ns, not significant; *p < .05; **p < .01; ***p < .001; ****p < .0001.
**FIGURE S2:** Methylation levels of individual markers ZNF582, SST, miR124‐2 across vulvar disease categories. Number of valid cases for each multiplex is reported in the legend. ns, not significant; *p < .05; **p < .01; ***p < .001; ****p < .0001.
**FIGURE S3:** HPV16 phylogenetic trees from patients diagnosed with HSIL and VSCC lesions. (A) LCR and E6 HPV16 nucleotide sequences. (B) Full LCR nucleotide sequences. (C) Full E6 HPV16 nucleotide sequences. The maximum likelihood phylogenetic tree was inferred from the alignment of 35 sequences with PhyML, with the GTR+G nucleotide substitution model and 1000 bootstraps re‐sampling.

## Data Availability

The data generated in this study are available upon reasonable request from the corresponding author.

## References

[1] Thuijs NB , van Beurden M , Duin S , et al. High‐grade vulvar intraepithelial neoplasia: comprehensive characterization and long‐term vulvar carcinoma risk. Histopathology. 2024;84:301‐314. 37726173 10.1111/his.15050

[2] Halec G , Alemany L , Quiros B , et al. Biological relevance of human papillomaviruses in vulvar cancer. Mod Pathol. 2017;30:549‐562. 28059099 10.1038/modpathol.2016.197

[3] Clifford GM , Tenet V , Georges D , et al. Human papillomavirus 16 sub‐lineage dispersal and cervical cancer risk worldwide: whole viral genome sequences from 7116 HPV16‐positive women. Papillomavirus Res. 2019;7:67‐74. 30738204 10.1016/j.pvr.2019.02.001 PMC6374642

[4] Cornet I , Gheit T , Franceschi S , et al. Human papillomavirus type 16 genetic variants: phylogeny and classification based on E6 and LCR. J Virol. 2012;86:6855‐6861. 22491459 10.1128/JVI.00483-12 PMC3393538

[5] Cornet I , Gheit T , Iannacone MR , et al. HPV16 genetic variation and the development of cervical cancer worldwide. Br J Cancer. 2013;108:240‐244. 23169278 10.1038/bjc.2012.508 PMC3553516

[6] Burk RD , Harari A , Chen Z . Human papillomavirus genome variants. Virology. 2013;445:232‐243. 23998342 10.1016/j.virol.2013.07.018 PMC3979972

[7] Dasgupta S , Ewing‐Graham PC , Swagemakers SMA , et al. Precursor lesions of vulvar squamous cell carcinoma—histology and biomarkers: a systematic review. Crit Rev Oncol Hematol. 2020;147:102866. 32058913 10.1016/j.critrevonc.2020.102866

[8] Steenbergen RDM , Snijders PJF , Heideman DAM , Meijer CJLM . Clinical implications of (epi)genetic changes in HPV‐induced cervical precancerous lesions. Nat Rev Cancer. 2014;14:395‐405. 24854082 10.1038/nrc3728

[9] De Strooper LM , Meijer CJ , Berkhof J , et al. Methylation analysis of the FAM19A4 gene in cervical scrapes is highly efficient in detecting cervical carcinomas and advanced CIN2/3 lesions. Cancer Prev Res Phila. 2014;7:1251‐1257. 25281488 10.1158/1940-6207.CAPR-14-0237

[10] Verlaat W , Snoek BC , Heideman DAM , et al. Identification and validation of a 3‐gene methylation classifier for HPV‐based cervical screening on self‐samples. Clin Cancer Res. 2018;24:3456‐3464. 29632006 10.1158/1078-0432.CCR-17-3615 PMC6053041

[11] van der Zee RP , Richel O , van Noesel CJM , et al. Host cell deoxyribonucleic acid methylation markers for the detection of high‐grade anal intraepithelial neoplasia and anal cancer. Clin Infect Dis. 2019;68:1110‐1117. 30060049 10.1093/cid/ciy601 PMC6424081

[12] Thuijs NB , Berkhof J , Özer M , et al. DNA methylation markers for cancer risk prediction of vulvar intraepithelial neoplasia. Int J Cancer. 2021;148:2481‐2488. 33426639 10.1002/ijc.33459 PMC8048962

[13] Swarts DRA , Voorham QJM , van Splunter AP , et al. Molecular heterogeneity in human papillomavirus‐dependent and ‐independent vulvar carcinogenesis. Cancer Med. 2018;7:4542‐4553. 30030907 10.1002/cam4.1633 PMC6144162

[14] Voss FO , Thuijs NB , Duin S , et al. Clinical validation of methylation biomarkers for optimal detection of high‐grade vulvar intraepithelial neoplasia. Int J Cancer. 2023;153:783‐791. 37074263 10.1002/ijc.34537

[15] Thomas LK , Bermejo JL , Vinokurova S , et al. Chromosomal gains and losses in human papillomavirus‐associated neoplasia of the lower genital tract—a systematic review and meta‐analysis. Eur J Cancer. 2014;50:85‐98. 24054023 10.1016/j.ejca.2013.08.022

[16] Hanahan D , Weinberg RA . Hallmarks of cancer: the next generation. Cell. 2011;144:646‐674. 21376230 10.1016/j.cell.2011.02.013

[17] Wilting SM , Steenbergen RD , Tijssen M , et al. Chromosomal signatures of a subset of high‐grade premalignant cervical lesions closely resemble invasive carcinomas. Cancer Res. 2009;69:647‐655. 19147580 10.1158/0008-5472.CAN-08-2478

[18] Bodelon C , Vinokurova S , Sampson JN , et al. Chromosomal copy number alterations and HPV integration in cervical precancer and invasive cancer. Carcinogenesis. 2016;37:188‐196. 26660085 10.1093/carcin/bgv171 PMC4834967

[19] Wilting SM , Steenbergen RDM . Molecular events leading to HPV‐induced high grade neoplasia. Papillomavirus Res. 2016;2:85‐88. 29074190 10.1016/j.pvr.2016.04.003 PMC5886901

[20] Gagne SE , Jensen R , Polvi A , et al. High‐resolution analysis of genomic alterations and human papillomavirus integration in anal intraepithelial neoplasia. JAIDS. 2005;40:182‐189. 16186736 10.1097/01.qai.0000179460.61987.33

[21] Huang FY , Kwok YKY , Lau ET , Tang MHY , Ng TY , Ngan HYS . Genetic abnormalities and HPV status in cervical and vulvar squamous cell carcinomas. Cancer Genet Cytogenet. 2005;157:42‐48. 15676146 10.1016/j.cancergencyto.2004.06.002

[22] Lavorato‐Rocha AM , Akagi EM , de Melo Maia B , et al. An integrative approach uncovers biomarkers that associate with clinically relevant disease outcomes in vulvar carcinoma. Mol Cancer Res. 2016;14:720‐729. 27170308 10.1158/1541-7786.MCR-15-0366

[23] Han M‐R , Shin S , Park H‐C , et al. Mutational signatures and chromosome alteration profiles of squamous cell carcinomas of the vulva. Exp Mol Med. 2018;50:e442. 29422544 10.1038/emm.2017.265 PMC5903820

[24] Jary A , Kim Y , Rozemeijer K , et al. Accurate detection of copy number aberrations in FFPE samples using the mFAST‐SeqS approach. Exp Mol Pathol. 2024;137:104906. 38820761 10.1016/j.yexmp.2024.104906

[25] Thuijs NB , van Beurden M , Bruggink AH , Steenbergen RDM , Berkhof J , Bleeker MCG . Vulvar intraepithelial neoplasia: incidence and long‐term risk of vulvar squamous cell carcinoma. Int J Cancer. 2021;148:90‐98. 32638382 10.1002/ijc.33198 PMC7689827

[26] Pakdel F , Farhadi A , Pakdel T , et al. The frequency of high‐risk human papillomavirus types, HPV16 lineages, and their relationship with p16(INK4a) and NF‐κB expression in head and neck squamous cell carcinomas in southwestern Iran. Braz J Microbiol. 2021;52:195‐206. 33169334 10.1007/s42770-020-00391-1 PMC7966695

[27] Belic J , Koch M , Ulz P , et al. Rapid identification of plasma DNA samples with increased ctDNA levels by a modified FAST‐SeqS approach. Clin Chem. 2015;61:838‐849. 25896989 10.1373/clinchem.2014.234286

[28] Akoglu H . User's guide to correlation coefficients. Turk J Emerg Med. 2018;18:91‐93. 30191186 10.1016/j.tjem.2018.08.001 PMC6107969

[29] Shen H , Laird PW . Interplay between the cancer genome and epigenome. Cell. 2013;153:38‐55. 23540689 10.1016/j.cell.2013.03.008 PMC3648790

[30] Akhoundova D , Rubin MA . Clinical application of advanced multi‐omics tumor profiling: shaping precision oncology of the future. Cancer Cell. 2022;40:920‐938. 36055231 10.1016/j.ccell.2022.08.011

[31] Gao Y , Widschwendter M , Teschendorff AE . DNA methylation patterns in normal tissue correlate more strongly with breast cancer status than copy‐number variants. EBioMedicine. 2018;31:243‐252. 29735413 10.1016/j.ebiom.2018.04.025 PMC6013931

[32] Engqvist H , Parris TZ , Biermann J , et al. Integrative genomics approach identifies molecular features associated with early‐stage ovarian carcinoma histotypes. Sci Rep. 2020;10:7946. 32409713 10.1038/s41598-020-64794-8 PMC7224294

[33] Ushijima T , Clark SJ , Tan P . Mapping genomic and epigenomic evolution in cancer ecosystems. Science. 2021;373:1474‐1479. 34554797 10.1126/science.abh1645

[34] Nam AS , Chaligne R , Landau DA . Integrating genetic and non‐genetic determinants of cancer evolution by single‐cell multi‐omics. Nat Rev Genet. 2021;22:3‐18. 32807900 10.1038/s41576-020-0265-5 PMC8450921

[35] Nicolás‐Párraga S , Gandini C , Pimenoff VN , et al. HPV16 variants distribution in invasive cancers of the cervix, vulva, vagina, penis, and anus. Cancer Med. 2016;5:2909‐2919. 27654117 10.1002/cam4.870 PMC5083745

[36] Mirabello L , Yeager M , Cullen M , et al. HPV16 sublineage associations with histology‐specific cancer risk using HPV whole‐genome sequences in 3200 women. JNCI J Natl Cancer Inst. 2016;108(9):djw100. doi:10.1093/jnci/djw100 27130930 PMC5939630

[37] Verlaat W , Van Leeuwen RW , Novianti PW , et al. Host‐cell DNA methylation patterns during high‐risk HPV‐induced carcinogenesis reveal a heterogeneous nature of cervical pre‐cancer. Epigenetics. 2018;13:769‐778. 30079796 10.1080/15592294.2018.1507197 PMC6224221

[38] Trietsch MD , Nooij LS , Gaarenstroom KN , van Poelgeest MIE . Genetic and epigenetic changes in vulvar squamous cell carcinoma and its precursor lesions: a review of the current literature. Gynecol Oncol. 2015;136:143‐157. 25448458 10.1016/j.ygyno.2014.11.002

[39] Bryndorf T , Kirchhoff M , Larsen J , et al. The most common chromosome aberration detected by high‐resolution comparative genomic hybridization in vulvar intraepithelial neoplasia is not seen in vulvar squamous cell carcinoma. Cytogenet Genome Res. 2004;106:43‐48. 15218240 10.1159/000078559

[40] Micci F , Panagopoulos I , Haugom L , et al. Genomic aberration patterns and expression profiles of squamous cell carcinomas of the vulva. Genes Chromosomes Cancer. 2013;52:551‐563. 23404381 10.1002/gcc.22053

[41] Lando M , Fjeldbo CS , Wilting SM , et al. Interplay between promoter methylation and chromosomal loss in gene silencing at 3p11‐p14 in cervical cancer. Epigenetics. 2015;10:970‐980. 26291246 10.1080/15592294.2015.1085140 PMC4844207

[42] Kinde I , Papadopoulos N , Kinzler KW , Vogelstein B . FAST‐SeqS: a simple and efficient method for the detection of aneuploidy by massively parallel sequencing. PLoS One. 2012;7:e41162. 22815955 10.1371/journal.pone.0041162 PMC3399813

[43] Belic J , Koch M , Ulz P , et al. mFast‐SeqS as a monitoring and pre‐screening tool for tumor‐specific aneuploidy in plasma DNA. In: Gahan PB , Fleischhacker M , Schmidt B , eds. Circulating Nucleic Acids in Serum and Plasma—CNAPS IX. Springer; 2016:147‐155. 10.1007/978-3-319-42044-8_28 27753036

[44] Mendelaar PAJ , Robbrecht DGJ , Rijnders M , et al. Genome‐wide aneuploidy detected by mFast‐SeqS in circulating cell‐free DNA is associated with poor response to pembrolizumab in patients with advanced urothelial cancer. Mol Oncol. 2022;16:2086‐2097. 35181986 10.1002/1878-0261.13196 PMC9120908

[45] Isebia KT , Mostert B , Deger T , et al. mFast‐SeqS‐based aneuploidy score in circulating cell‐free DNA is a prognostic biomarker in prostate cancer. Mol Oncol. 2023;17:1898‐1907. 37178439 10.1002/1878-0261.13449 PMC10483599

[46] van Harten AM , Poell JB , Buijze M , et al. Characterization of a head and neck cancer‐derived cell line panel confirms the distinct TP53‐proficient copy number‐silent subclass. Oral Oncol. 2019;98:53‐61. 31541927 10.1016/j.oraloncology.2019.09.004 PMC7372097

