# Supplementary material for: DNA methylation and copy number alterations in the progression of HPV‐associated high‐grade vulvar intraepithelial lesion
Source: Int J Cancer. 2025 Feb 12;156(10):1926–35. doi: 10.1002/ijc.35366 (PMC11924301; doi:10.1002/ijc.35366)
Supplement: Supplementary file 1 — TABLE S1: Primers sequences for the amplification of HPV16 E6 and LCR viral genome regions. TABLE S2: PCR protocols for the amplification of HPV16 E6 and LCR viral genome regions. TABLE S3: Number of total reads, total mapped reads, and median number of mapped reads per chromosomal arm*, per sample obtained after mFAST‐SeqS. Reads were mapped to the human genome hg19. *mFAST‐SeqS is a low‐resolution technique which performs analysis at chromosomal arm level. TABLE S4: HPV16 variants in patients diagnosed with HSIL and VSCC lesions. FIGURE S1: Total number of CNAs per sample across vulvar disease categories. Number of sequenced samples is reported in the legend. In the boxplots, hinges correspond to the 1st and 3rd quantiles, whiskers “min” and “max” correspond to 1.5*IQR, horizontal lines indicate the median. Triangles represent HSIL patients who developed VSCC during follow‐up. ns, not significant; *p < .05; **p < .01; ***p < .001; ****p < .0001. FIGURE S2: Methylation levels of individual markers ZNF582, SST, miR124‐2 across vulvar disease categories. Number of valid cases for each multiplex is reported in the legend. ns, not significant; *p < .05; **p < .01; ***p < .001; ****p < .0001. FIGURE S3: HPV16 phylogenetic trees from patients diagnosed with HSIL and VSCC lesions. (A) LCR and E6 HPV16 nucleotide sequences. (B) Full LCR nucleotide sequences. (C) Full E6 HPV16 nucleotide sequences. The maximum likelihood phylogenetic tree was inferred from the alignment of 35 sequences with PhyML, with the GTR+G nucleotide substitution model and 1000 bootstraps re‐sampling. [file IJC-156-1926-s001.pdf]

Supplementary Material for

DNA methylation and copy number alterations in the progression of HPV-associated high-grade vulvar intraepithelial lesion

Flavia Runello, Aude Jary, Sylvia Duin, Yongsoo Kim, Kahren van Eer, Féline O. Voss, Nikki B. Thuijs, Maaike C.G. Bleeker, Renske D.M. Steenbergen

Table of contents

Supplementary Table 1. Primers sequences for the amplification of HPV16 E6 and LCR viral genome regions……………………………………………………………………………………………………..2

Supplementary Table 2. PCR protocols for the amplification of HPV16 E6 and LCR viral genome regions……………………………………………………………………………………………………..3

Supplementary Table 3. Number of total reads, total mapped reads, and median number of mapped reads per chromosomal arm, per sample obtained after mFAST-SeqS…….4

Supplementary Table 4. HPV16 variants in patients diagnosed with HSIL and VSCC lesions…………6

Supplementary Figure legends.……………………………………………………………………………7

Supplementary Figure 1. Total number of CNAs per sample across vulvar disease categories………………………………………………………………………………………………………8

Supplementary Figure 2. Methylation levels of individual markers *ZNF582, SST, miR124-2* across vulvar disease categories……………………………………………………………………………………9

Supplementary Figure 3. HPV16 phylogenetic trees from patients diagnosed with vulvar lesions……………………………………………………………………………………………………..10

Supplementary Table 1. Primers sequences for the amplification of HPV16 E6 and LCR viral genome regions.

Names	Sequences (5′ - 3′)	Nucleotide positions	Product size (bp)
16E6-1F-outer	ttgaaccgaaaccggttagt	46–65	393
16E6-2R-outer	ggacacagtggcttttgaca	419–438	
16E6-1F-inner	ttgaaccgaaaccggttagt	46–65	211
16E6-1R-inner	gcataaatcccgaaaagcaa	237–256	
16E6-2F-inner	gcaacagttactgcgacgtg	205–244	234
16E6-2R-inner	ggacacagtggcttttgaca	419–438	
16E6-2F-outer	gcaacagttactgcgacgtg	205–224	386
16E6-3R-outer	tcatgcaatgtaggtgtatctcc	568–590	
16E6-3F-inner	cagcaatacaacaaaccgttg	371–391	220
16E6-3R-inner	tcatgcaatgtaggtgtatctcc	568–590	
16LCR-1F-outer	gaaaacgaaaagctacaccca	7084–7104	497
16LCR-2R-outer	gtgcaggtcaggaaaacag	7562–7580	
16LCR-1F-inner	gaaaacgaaaagctacaccca	7084–7104	285
16LCR-1R-inner	caatgaataaccacaacacaatta	7345–7368	
16LCR-2F-inner	gcttgtgtaactattgtgtcatg	7289–7311	292
16LCR-2R- inner	gtgcaggtcaggaaaacag	7562–7580	
16LCR-3F-outer	acttgtacgtttcctgcttg	7525–7544	483
16LCR-4R-outer	tgcagttctcttttggtgc	85–103	
16LCR-3F-inner	acttgtacgtttcctgcttg	7525–7544	350
16LCR-3R-inner	gtgtaacccaaaatcggtttgc	7853–7874	
16LCR-4F-inner	gtcaccctagttcatacatga	7777–7797	231
16LCR-4R-inner	tgcagttctcttttggtgc	85–103	

Supplementary Table 2. PCR protocols for the amplification of HPV16 E6 and LCR viral genome regions.

1. First PCR (Outer primers)

The amplification of the 4 outer regions (16E6 1F-2R, 16E6 2F-3R, 16LCR 1F-2R, 16LCR 3F-4R) was performed with the AmpliTaq DNA Pol (Applied Biosystems^{TM}) according to the following protocol:

	Concentration	1x (µl)	PCR program			Cycles
10X Buffer	1X	2.5				
10 mM dNTP Mix	0.2 mM each	0.5	Holding stage	2 min	95ºC	1x
25 mM MgCl2 (with Buffer II)	1.5 mM	1.5				
10 uM forward primer	0.2 µM	0.5	Cycling stage	15 sec	95ºC	
10 uM reverse primer	0.2 µM	0.5		30 sec	55ºC	45x
AmpliTaq DNA Pol (5U/uL)	1.25 / 50 µL rxn	0.13		45 sec	72ºC	
Nuclease-free water	-	16.8				
DNA sample		2.5	Final extension	5 min	72ºC	1x
**Total**		25		forever	8ºC	

2. Nested PCR (Inner primers)

The amplification of the 7 inner regions (16E6 1F-1R, 16E6 2F-2R, 16E6 3F-3R, 16LCR 1F-1R, 16LCR 2F-2R, 16LCR 3F-3R, 16LCR 4F-4R) was performed with the AmpliTaq DNA Pol (Applied Biosystems^{TM}) according to the following protocol:

	Concentration	1x (µl)	PCR program			Cycles
10X Buffer	1X	2.5				
10 mM dNTP Mix	0.2 mM each	0.5	Holding stage	2 min	95ºC	1x
25 mM MgCl2 (with Buffer II)	1.5 mM	1.5				
10 uM forward primer	0.2 µM	0.5	Cycling stage	15 sec	95ºC	
10 uM reverse primer	0.2 µM	0.5		30 sec	55ºC	45x
AmpliTaq DNA Pol (5U/uL)	1.25 / 50 µL rxn	0.13		30 sec	72ºC	
Nuclease-free water	-	18.4				
PCR product (first PCR)		1	Final extension	5 min	72ºC	1x
**Total**		25		forever	8ºC	

Supplementary Table 3. Number of total reads, total mapped reads, and median number of mapped reads per chromosomal arm*, per sample obtained after mFAST-SeqS. Reads were mapped to the human genome hg19. *mFAST-SeqS is a low-resolution technique which performs analysis at chromosomal arm level.

Samples	Total reads	Total mapped reads	Median mapped reads/ chromosomal arm (n)
1	230833	189934	3571.5
2	179881	142882	2686
3	145269	117585	2203.5
4	185668	155207	3000
5	115766	93305	1788.5
6	315040	265471	4918.5
7	160207	128311	2329.5
8	155104	126053	2323
9	170769	116837	4652.5
10	179604	122624	6975.5
11	136294	110914	2071
12	117318	100579	1854.5
13	139791	99479	3968.5
14	114711	93026	1768.5
15	130573	105487	2344
16	103621	89069	1637.5
17	178062	119936	4048.5
18	143711	120864	2276
19	130573	105487	2006.5
20	122142	103289	1989.5
21	148063	125627	2425.5
22	128280	107528	1951.5
23	76738	62849	1190
24	120731	101759	1884
25	80653	66939	1271.5
26	133741	95202	1773
27	203773	162272	2930
28	192097	130647	2343.5
29	104803	86309	1564
30	104854	84210	1606
31	132266	104716	1910.5
32	153192	121572	2312
33	207016	172321	3239
34	123557	100221	1837
35	179903	150860	2854.5
36	76105	60181	1118.5
37	275578	235376	4363
38	144272	120511	2222.5
39	164360	110043	2070.5

40	80781	65958	1238
41	94954	77473	1401.5
42	163010	109392	2028
43	74409	60826	1122.5
44	85516	69623	1272.5
45	116522	99915	1768.5
46	119481	95615	1741.5
47	173555	145982	2655.5
48	132056	105239	1897
49	195723	130332	2392.5
50	201337	167824	3170.5
51	240520	199936	3625.5
52	118935	94262	1733.5
53	129694	102153	1875
54	150611	126373	2352
55	237048	200057	3457.5
56	126145	100466	1792
57	140388	112107	1976
58	132433	107045	1952
59	152444	124328	2232
60	178918	147151	2707
61	88886	73250	1326
62	253257	202536	3646
63	62420	51854	913.5
64	164545	137287	2415
65	222326	179903	3157
66	119520	98797	1776
67	195353	162117	3007
68	191499	156284	2700
69	317794	260504	4333.5
70	174947	117654	6993
71	134115	89104	11428
72	134039	107878	1958
73	121340	79580	13302
74	99890	80513	1435
75	119997	101070	1436
76	114468	96126	1659
77	114144	91756	1690.5
78	151788	121619	2223
79	119255	94277	1619
80	98110	80617	1494.5
81	134956	111554	1906
82	133049	104710	1964

Supplementary Table 4. HPV16 variants in patients diagnosed with HSIL and VSCC lesions.

Samples	High risk HPV typing	Ct value	Sanger sequencing	Variants
20	HPV16	25.72	no amplification	-
23	HPV16	34.58	no amplification	-
24	HPV16	24.67	Full E6	A2
25	HPV16	26.45	Full E6 and LCR	D3
26	HPV16	20.23	Full E6 and LCR	A1
27	HPV16	28.72	no amplification	-
28	HPV16	20.94	Partial E6 and LCR	A
30	HPV16	25.9	Partial E6	C1
31	HPV16	24.8	Partial E6 and LCR	A
32	HPV16	18.6	Full E6 and LCR	A1
33	HPV16	25.57	Partial E6 and LCR	A2
34	HPV16	18.93	Full E6 and LCR	A1
35	HPV16	18.75	Full E6 and LCR	A1
36	HPV16	23.79	Full E6	A2
37	HPV16	25.35	Partial E6 and LCR	A1
39	HPV16	26.16	Partial E6 and LCR	A1
40	HPV16	20.4	Full E6	A2
41	HPV16	25.73	Full E6	A2
43	HPV16	27.06	no amplification	-
45	HPV16	31.69	Full E6	A
46	HPV16	19.26	Full E6	A
47	HPV16	23.15	Full E6 and LCR	A
48	HPV16	21.48	Full E6 and LCR	A1
49	HPV16	21.52	Full E6	A2
50	HPV16	24.74	Partial E6 and LCR	A2
51	HPV16 and other	21.72	Full E6	A2
52	HPV16	19.05	Full E6	A2
53	HPV16	18.62	Full E6 and LCR	A2
54	HPV16	24.68	Partial E6 and LCR	A1
55	HPV16	20.46	Full E6 and LCR	A2
56	HPV16	20.43	no amplification	-
57	HPV16	22.4	Full E6 and LCR	D3
58	HPV16	17.45	Full E6 and LCR	A2
59	HPV16	20.73	Partial E6 and LCR	A
60	HPV16	26.63	Full E6 and LCR	A1
61	HPV16	21.75	Full E6 and LCR	A1
62	HPV16	21.21	Full E6	A2
64	HPV16	16.74	Full E6	B
65	HPV16	unknown	Full E6 and LCR	A1
66	HPV16	23.56	Full E6	A
67	HPV16	20.61	Full E6 and LCR	A1
68	HPV16	26.99	Partial E6 and LCR	C1
69	HPV16	25.74	Partial E6 and LCR	A1
71	HPV16	30.3	no DNA left	-
72	HPV16	35.47	Full E6	A1
73	HPV16	23.01	Full E6 and LCR	A1
74	HPV16	24.56	Partial E6 and LCR	A2
75	HPV16	20.96	Full E6	A
76	HPV16	24.48	Full E6 and LCR	B
77	HPV16	24.73	Full E6	A
78	HPV16	unknown	no amplification	-
82	HPV16	23.02	no DNA left	-

Supplementary Figure 1. Total number of CNAs per sample across vulvar disease categories.

Number of sequenced samples is reported in the legend. In the boxplots, hinges correspond to the 1st and 3rd quantiles, whiskers “min” and “max” correspond to 1.5*IQR, horizontal lines indicate the median. Triangles represent HSIL patients who developed VSCC during follow-up.

ns, not significant; *P < .05; **P < .01; ***P < .001; ****P < .0001.

Supplementary Figure 2. Methylation levels of individual markers *ZNF582, SST, miR124-2* across vulvar disease categories.

Number of valid cases for each multiplex is reported in the legend.

ns, not significant; *P < .05; **P < .01; ***P < .001; ****P < .0001.

Supplementary Figure 3. HPV16 phylogenetic trees from patients diagnosed with HSIL and VSCC lesions.

A. LCR and E6 HPV16 nucleotide sequences.

B. Full LCR nucleotide sequences.

C. Full E6 HPV16 nucleotide sequences.

The maximum likelihood phylogenetic tree was inferred from the alignment of 35 sequences with PhyML, with the GTR+G nucleotide substitution model and 1000 bootstraps re-sampling.

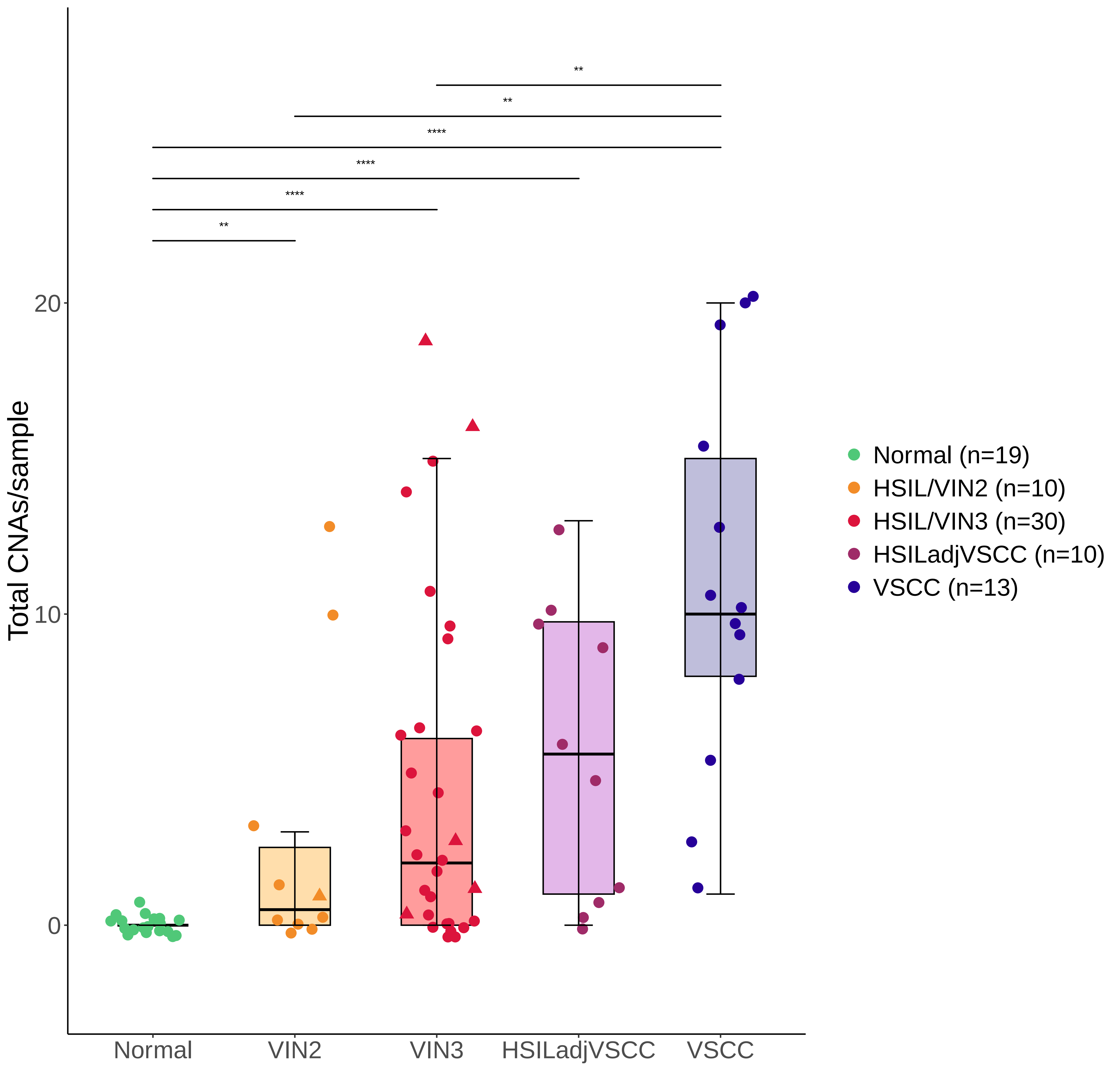

**
**
****
****
****
**
Total CNAs/sample
20
10
0
Normal
VIN2
VIN3
HSILadjVSCC
VSCC
Normal (n=19)
HSIL/VIN2 (n=10)
HSIL/VIN3 (n=30)
HSILadjVSCC (n=10)
VSCC (n=13)

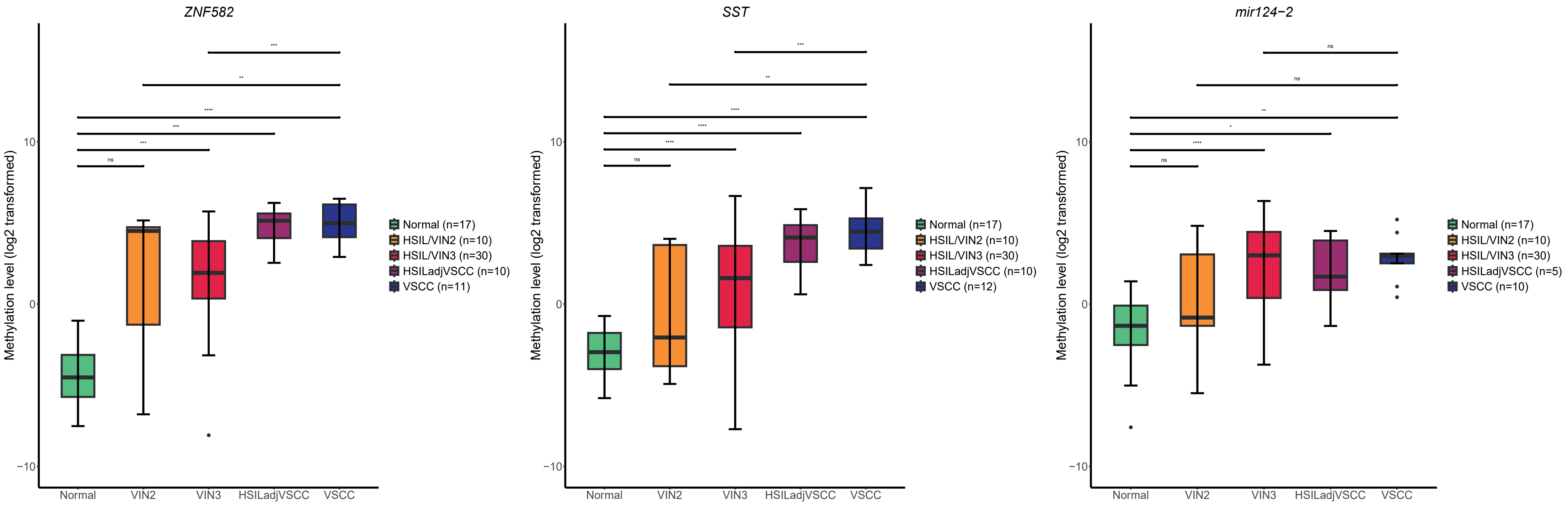
ZNF582
SST
mir124−2
Methylation level (log2 transformed)
10
0
−10
Normal
VIN2
VIN3
HSILadjVSCC
VSCC
ns
Normal (n=17)
HSIL/VIN2 (n=10)
HSIL/VIN3 (n=30)
HSILadjVSCC (n=10)
VSCC (n=11)
VSCC (n=12)
HSILadjVSCC (n=5)
VSCC (n=10)

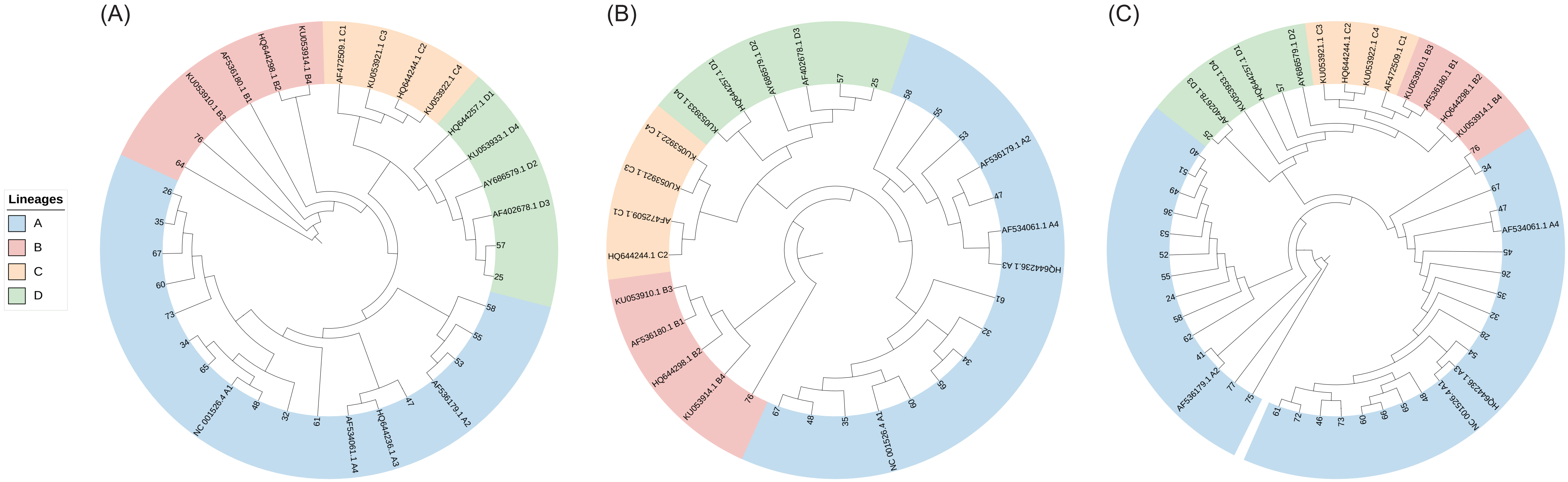
(A)
(B)
(C)
Lineages
A
B
C
D
NC 001526.4 A1
AF536179.1 A2
HQ644236.1 A3
AF534061.1 A4
AF536180.1 B1
HQ644298.1 B2
KU053910.1 B3
KU053914.1 B4
AF472509.1 C1
HQ644244.1 C2
KU053921.1 C3
KU053922.1 C4
HQ644257.1 D1
AY686579.1 D2
AF402678.1 D3
KU053933.1 D4
